# Recent advances in polymeric nanoparticles for the treatment of hepatic diseases

**DOI:** 10.3389/fphar.2025.1528752

**Published:** 2025-01-24

**Authors:** Feng Gao, Xuefei Feng, Xinyu Li

**Affiliations:** Clinical Laboratory of China-Japan Union Hospital, Jilin University, Changchun, China

**Keywords:** hepatic diseases, liver cancer, nanomaterials, polymeric nanoparticles, drug delivery

## Abstract

The liver performs crucial roles in energy metabolism, detoxification, and immune regulation. Hepatic diseases, including hepatitis, liver fibrosis, and liver cancer, have posed a significant threat to global health, emphasizing the critical need for the development of novel and effective treatment approaches. Nanotechnology, an emerging technology, has been extensively researched in medicine. Among the many types of nanomaterials, polymeric nanoparticles (NPs) are widely used in drug delivery systems. Compared to traditional therapies, they offer significant advantages in the treatment of liver disease by improving outcomes and reducing side effects. This review introduced the development of liver disease and discussed the application of natural polymers and synthetic polymers in their management. Furthermore, this paper reviewed the application of polymeric nanoparticles -mainly chitosan (CS), hyaluronic acid (HA), polyethylene glycol (PEG) and poly (lactic-co-glycolic acid) (PLGA)-in liver disease treatment, focusing on their use in various delivery systems for pure bioactive compounds of natural origin, drugs, nucleic acids, peptides, and others. Finally, the challenges and future perspectives of the NPs were discussed to provide guidance for further research directions, with the aim of promoting the clinical application of nanotherapeutics in treating hepatic diseases.

## 1 Introduction

The liver is one of the largest organs in the human body, playing crucial roles in energy metabolism, detoxification, and immune regulation (Nicole et al., 2019). Certain chemicals, drugs, food, and various infections (bacterial, fungal, or viral) might cause hepatic diseases such as hepatitis, liver fibrosis and liver cancer (Aydın and Akçalı, 2018; Sumeet et al., 2019; De Mattos et al., 2024). Hepatitis is characterized by widespread or patchy necrosis in liver inflammation, comprising acute and chronic forms. Acute hepatitis typically resolves spontaneously, while chronic hepatitis is characterized by persistent liver inflammation and hepatocellular necrosis lasting for at least 6 months, typically caused by hepatitis viruses. Hepatitis viruses primarily replicate in the liver, causing hepatocyte necrosis, oxidative stress, inflammatory responses, and immune activation. This triggers a cycle of liver cells injury and repair, alongside the activation of hepatic stellate cells (HSCs) and fibroblasts, which secrete collagen, cytokines, pro-fibrotic factors, and other extracellular matrix components, promoting excessive fibrosis. Severe fibrosis could lead to cirrhosis, liver cancer, and even death (Pei et al., 2023). Clinical treatment methods for hepatic diseases are diverse, including surgical interventions (hepatectomy and liver transplantation), ablation therapy, chemotherapy, radiotherapy, antiviral therapy, and immunotherapy. Despite significant advancements in the clinic, some challenges hinder the effective use of current hepatic disease treatments, such as a shortage of donor organs for liver transplants (Tapper and Parikh, 2023). Furthermore, some drugs are constrained in their therapeutic potential by factors such as limited distribution and bioavailability, low solubility, as well as potential toxicity and adverse effects (Yang et al., 2022). Nanomedicines could improve therapeutic efficacy and reduce adverse effects of drugs (e.g., pure bioactive compounds of natural origin, chemotherapeutic drugs, and nucleic acid-based medicines) as new drug delivery systems (DDS), thus providing a new paradigm to address the above challenges.

**FIGURE 1:**
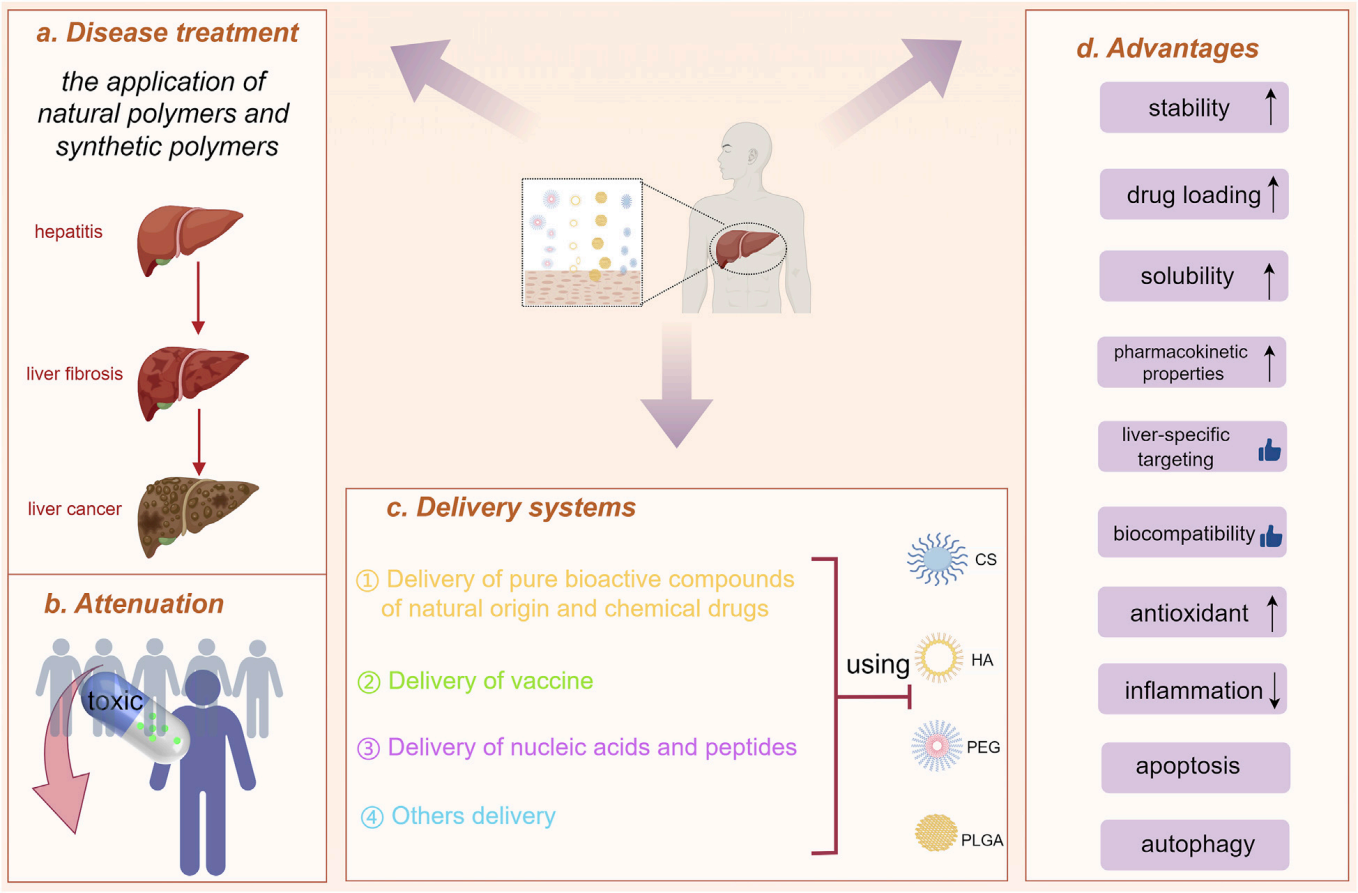
Polymeric nanoparticles (natural and synthetic polymers) used to treat liver diseases. **(A)** Disease treatment. **(B)** Attenuation. **(C)** The application of chitosan (CS), hyaluronic acid (HA), polyethylene glycol (PEG) and poly(lactic-co-glycolic acid) (PLGA) in liver disease treatment, focusing on their use in various delivery systems. **(D)** Advantages. (drawn by Figdraw, ID: UWPAY4434a).

**FIGURE 2:**
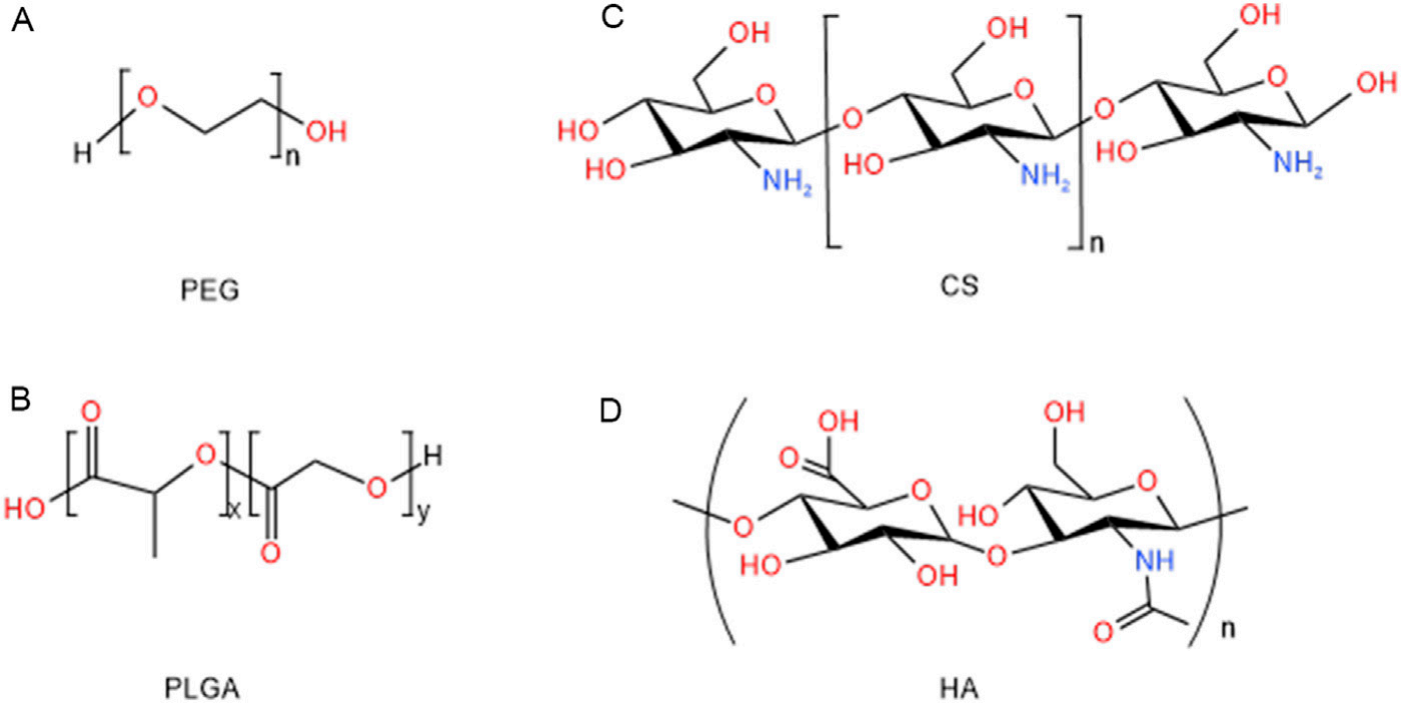
Structure of polymeric nanoparticles for the treatments of hepatic diseases: **(A)** PEG, **(B)** PLGA, **(C)** CS, **(D)** HA.

The explosive advancement of nanotechnology over the past few decades was significantly impacting the field of medicine. Nanomaterials have obvious advantages not only in improving pharmacokinetics, prolonging blood circulation time and reducing drug toxicity, but also in targeting drug delivery, slowing drug release, and improving drug solubility. Many nanomaterials and micromaterials, such as polymeric nanoparticles, polymeric micelles, inorganic nanoparticles, dendrimers, nanogels and solid lipid nanoparticles have been explored in hepatic diseases treatment (Paolo and Pietro, 2019). Their delivery methods are usually categorized as passive targeting and active targeting. Passive targeting utilized the properties of nanoparticles, such as composition, particle size, and charge, to enable targeted delivery of drugs to specific areas within the organism. Active targeting utilized specific ligands to bind to cellular receptors for drug delivery. Passive and active targeting strategies could significantly enhance drug accumulation at liver disease sites, control drug release, reverse drug resistance, and reduce side effects (Yuxin et al., 2024). Among these, polymeric nanoparticles and solid lipid nanoparticles have been widely used in drug delivery systems. However, solid lipid nanoparticles were constrained by limited stability and challenges in lipid functionalization. In contrast, polymeric nanoparticles have been the most widely used due to their advantages to encapsulate diverse bioactive compounds, protect the encapsulated drugs, and enable targeted delivery (Beach et al., 2024). In view of the importance and growing interest in this field, we presented various types of polymers being used as drug delivery systems for hepatitis, liver fibrosis and liver cancer in this review (Table 1). Futhermore, this paper reviewed the application of polymeric nanoparticles-mainly chitosan (CS), hyaluronic acid (HA), polyethylene glycol (PEG) and poly (lactic-co-glycolic acid) (PLGA)-in liver disease treatment, focusing on their use in various delivery systems for pure bioactive compounds of natural origin, drugs, nucleic acids, peptides, and others (Figures 1, 2). And we hope that will trigger some new ideas and stimulate more efforts to promote the widespread use of nanotechnology intreating hepatic diseases.

**TABLE 1:** Summary of the application of polymers in hepatitis, liver fibrosis and liver cancer.

Carriers	Functional component	Size	Cell lines/Animal models	Hepatic diseases	Ref.
CS	Betulinic acid	<200 nm	C57BL/6 mice, HepG2 cells, LX-2 cells, LO2 cells	Liver fibrosis	Wu et al. (2022)
CS	Green tea extract	∼200–250 nm	HepG2 cells, SD rats	Liver fibrosis	Safer et al. (2015)
CS	Sorafenib	212.4 ± 59.7 nm; 24.1 ± 10.2 nm; 16.6 ± 3.0 nm; 9.2 ± 2.0 nm	HepG2 cells, HDFa	Liver cancer	Albalawi et al. (2023)
CS	Deoxycholic acid, folic acid	126 nm	Specific pathogen-free mice, MCF-7 cells, H22 cells	Liver cancer	Li et al. (2018)
CS	Compound k	170–200 nm	HepG2 cells	Liver cancer	Zhang et al. (2018)
HA	Astaxanthin	210–500 nm	SD rats	Liver fibrosis	Wu et al. (2018)
HA	Bilirubin	155 ± 83.41 nm	HepG2 cells, AML12 cells, LX-2 cells, C57BL/6J mice	Liver fibrosis	Shinn et al. (2024)
HA/PLGA	Doxorubicin	27 μm	HepG2 cells, McA-RH7777	Liver cancer	Lee et al. (2018)
HA	Camptothecin	90 nm	MCF-7, balb/c mice	Liver cancer	Chen et al. (2016b)
Alginate	MSA-2	—	Lucifere-H22 cells, H22 cells, RAW 264.7 cells, C1249	Liver cancer	Feng et al. (2024)
Cellulose	Dipterocarpol	1.38 ± 0.079 μm, 1.71 ± 0.14 μm	HepG2 cells	Liver cancer	Piman et al. (2024)
Gelatin	Doxorubicin	100 nm	Tumor cells	Liver cancer	Wang et al. (2024)
Dextran	siSnail2	200 nm	A549 cells, HepG2 cells, BALB/c nude mice	Liver cancer	Hu et al. (2024b)
γ-PGA	Hepatitis B surface antigen	265.3 ± 7.28 nm	C57BL/6J mice	Hepatitis	Haigang et al. (2018)
γ-PGA	Gambogic acid	50 nm	HepG2 cells, LO2 cells, ICR mice	Liver cancer	Han et al. (2008)
PCL	Chrysin	94.57 ± 13.40 nm	HepG2 cells	Liver cancer	Alshetaili et al. (2023)
PEG-PCL	Vitamin A-derivative	90.3 nm	Rat primary HSCs cells, hepatocytes, HSC-T6 cells, LX-2 cells	Liver fibrosis	Li X. et al. (2023)
PEG	Oncolytic herpes simplex virus	247.2 nm	Hepa1-6 cells, C57BL/6 mice	Liver cancer	Liang et al. (2023)
PEG	Sorafenib	115.1 nm	HepG2 cells	Liver cancer	Xiaolong et al. (2018)
PEG-PLGA	Aminoethyl anisamide	135 nm	C57BL/6 mice, Hepa1-6 cells	Liver cancer	Meng et al. (2023)
PEG-PLGA	Cryptotanshinone	144.7 ± 6.53 nm	BALB/c nude mice, HepG2 cells	Liver cancer	Nie et al. (2020)
PEG-PLGA	Cantharidin	25.32 ± 1.25 nm	HepG2 cells, LO2 cells	Liver cancer	Yao et al. (2020)
PLGA	Adefovir	<60 and 350 μm	Adult albino male rats	Hepatitis	GutAyoub et al. (2018)
PLGA	Spleen tyrosine kinase inhibitor R406	159.7 nm	RAW 264.7 cells, C57BL/6 mice	Liver fibrosis	Kurniawan et al. (2018)
PLGA	Ferulic acid	160 ± 7.1 nm	HSC-T6 cells, Raw 264.7 cells, C57BL/6 mice	Liver fibrosis	Hu et al. (2024a)
PLGA	Imatinib	125 nm	BALB/c mice	Liver fibrosis	Siapoush et al. (2023)
PLGA	R848	162.6 nm	LO2 cells, Hepa1-6 cells, HepG2 cells, C57BL/6 mice	Liver cancer	Liao et al. (2024)
PLGA	Sorafenib	248 nm	HepG2 cells	Liver cancer	Caputo et al. (2023)
PLGA	Paclitaxel	308–369.5 nm	HepG2 cells, Huh-7 cells, SD rats	Liver cancer	Mandal et al. (2018)
PAMAM	Sorafenib	—	Wistar rats	Liver fibrosis	Shafie et al. (2019)
PHPMA	5-fluorouracil	174.20 ± 3.59 nm	Bel-7402, nude mice	Liver cancer	Chen et al. (2022)
Hydroxyapatite	As_2_O_3_	67.7 nm	BEL-7402 cells	Liver cancer	Xue et al. (2020)

SD rats, Sprague Dawley rats; C1249, bonemarrow-dendritic cells; γ-PGA, poly-γ-glutamic acid; R848, resiquimod; PAMAM, poly (2-hydroxypropyl) methacrylamide; Bel-7402, Human hepatoma cells.

## 2 Polymeric nanoparticles

Polymeric nanoparticles have been employed in a range of traditional applications, including the manufacture of catheters, syringes, and implants for bone, cartilage, skin tissue, and blood vessels. In 1969, Speiser and his team first introduced the concept of polymeric nanoparticles for drug delivery (Shabir et al., 2017). Nowadays, numerous polymeric nanoparticles have been engineered for the targeted delivery of therapeutic agents, including vaccines, pure bioactive compounds of natural origin, nucleic acids, and small molecules, to the liver for the treatment of hepatic diseases. The focus of these advancements is on polymeric nanoparticles that exhibit favorable hydrophilicity, stability within the hematological system, protection of nucleic acids, and efficient endosomal/lysosomal escape, and appropriate surface charge for optimized drug delivery systems. Polymeric nanoparticles were typically divided into two main types according to their source: natural polymers and synthetic polymers. Natural polymers, known for their biocompatibility and biodegradability, were particularly suited for drug delivery and tissue engineering applications. In contrast, synthetic polymers, engineered to possess specific mechanical, chemical, and degradation properties, offered enhanced versatility for a wide range of biomedical and industrial uses (Galina et al., 2024).

### 2.1 Natural polymers

Natural polymers derive from biological sources such as plants, animals, or microorganisms. They are components of biological systems responsible for performing various essential functions (Bailey et al., 2023). There were many natural polymers frequently utilized for the development of polymeric nanoparticles, such as CS, HA, alginate, collagen, gelatin and cellulose.

CS, a linear β-1, 4-D-glucosamine, is a natural polysaccharide obtained from the deacetylation of chitin. It is the second most common natural polysaccharide in the world, after cellulose (Antonino et al., 2017). CS is non-toxicity and has no side effects, along with good moisturizing and adsorption properties. The United States. FDA has approved that CS is safe in the use of foods and drugs (Rizeq et al., 2019; Wang et al., 2020). CS has varied structures, sizes, and charges, and its chemical functional groups can be easily modified to meet the needs of specific applications and different modes of drug delivery (Rashki et al., 2021). CS has frequently been employed for the design of drug delivery systems due to its biocompatibility, natural degradability, mucosal adsorption, and other physicochemical properties, as well as its ability to demonstrate biological effects, including hypolipidemic, hypoglycemic, antioxidant, anti-inflammatory, antimicrobial, antibacterial, and immunomodulatory effects (Matalqah et al., 2020; Vohra et al., 2021). Meanwhile, CS has been used as a nano-adjuvant for vaccine and a carrier for drug delivery systems in liver disease.

HA is a glycosaminoglycan essential to the extracellular matrix (ECM). It is synthesized by hyaluronan synthases at the inner surface of the plasma membrane and is broken down through enzymatic hydrolysis of its b-1,4-glycosidic bonds by hyaluronidase (HAase) (Salih et al., 2024). It is found in large quantities in the vitreous humor, umbilical cord, dermis, synovial fluid, and heart valves. Approximately 80%–90% of HA is catabolized by the liver, while the remaining 10% is excreted via the kidneys *in vivo* (Saha and Rai, 2021; Matsumoto et al., 2022). Commercially, HA existed in three different molecular weight forms, high molecular weight (10,000 kD), medium molecular weight (500–800 kD) and low molecular weight (160–240 kD) HA. By modifying the molecular structure of HA, it was possible to create derivatives that were more resistant to mechanical and chemical forces while maintaining compatibility and biodegradability in biological environments. High molecular weight HA primarily exhibited immunosuppressive and anti-angiogenic properties, whereas medium and low molecular weight HA possessed pro-angiogenic and anti-apoptotic effects, induced the synthesis of heat shock proteins (HSP), and acted as effective immunostimulants (Zhu et al., 2020). The related literatures demonstrated that HA was non-toxic, biocompatible, and non-allergenic, promoting wound healing, tissue regeneration, anti-wrinkle effects, and anti-inflammatory responses. These findings might have implications for cancer prognosis (Ahmadian et al., 2019). Therefore, it was widely used in several medical fields, including orthopedics, dermatology, plastic surgery and cardiovascular surgery. In summary, HA’s diverse properties and versatile applications make it a valuable component in the development of therapeutic strategies for hepatic diseases and beyond.

Alginate is a natural polysaccharide primarily derived from brown algae, such as *Laminaria*, *Ascophyllum*, and *Macrocystis*. It has three advantages. First, the biodegradation of alginate under physiological conditions was demonstrated to occur via two distinct mechanisms: enzymatic cleavage by alginate lyase and acidic hydrolysis. Furthermore, modification of the chemical structure of alginate with functional groups or targeting ligands had the potential to enhance a number of properties, including stability, drug loading and liver-specific targeting (Piotr et al., 2021). And alginate readily formed gels in the presence of divalent cations like calcium ions (Ca^2⁺^), which facilitated the encapsulation of drugs for sustained or controlled release (Cassandra et al., 2022).

Collagen, the primary structural protein in connective tissues, was shown to enhance liver tissue repair by promoting hepatocyte adhesion, migration, and proliferation. It could deliver antifibrotic drugs directly to the liver, where it helped remodel the extracellular matrix and reduce fibrosis. Due to its structural similarity to the hepatic extracellular matrix, it tended to accumulate preferentially in liver tissue, thereby enhancing the efficiency of targeted drug delivery to the liver (Leora et al., 2019).

Gelatin, derived from collagen, a major component of the hepatic extracellular matrix, exhibited excellent biocompatibility. This property minimized immune responses, making it an ideal candidate for liver-targeted drug delivery. Gelatin was capable of encapsulating various therapeutic agents, such as anti-inflammatory, antifibrotic, and anticancer drugs (Yutao et al., 2024). In acidic lysosomal environments, gelatin degraded, enabling controlled drug release at the site of liver injury. Due to its water solubility, gelatin often required cross-linking during nanoparticle formulation to enhance stability and control release (Morán et al., 2015).

Cellulose is a polysaccharide found in plants cell walls (Pedersen et al., 2023). It exhibited both hydrophilic and hydrophobic properties, making them suitable for a wide range of biomedical applications. To enhance liver-specific drug delivery,it was functionalized with targeting ligands, a strategy that significantly improved their ability to selectively accumulate in hepatic tissues. This targeted delivery approach proved particularly valuable in the treatment of hepatic diseases, such as hepatitis and liver cancer, where localized drug release was essential for maximizing therapeutic efficacy while minimizing systemic side effects (Ansar et al., 2021).

### 2.2 Synthetic polymers

Synthetic polymers, which could be easily adjusted in terms of monomer ratios, molecular weights, and chemical bonding, were characterized by their diversity. Due to their unique physicochemical properties, drugs could be efficiently encapsulated or conjugated by synthetic polymers, allowing for specific targeting of liver tissue and providing extended drug release at liver sites (Mohammed and Naif, 2022). Common synthetic polymers used in nanoparticle fabrication included PEG, PLGA, polycaprolactone (PCL), polymethyl methacrylate (PMMA), and others (Table 1). In recent years, the interest in synthetic polymers has grown exponentially due to their superiority. Researchers have made several adjustments to develop effective nanoparticles, including particle size, zeta-potential, size distribution, shapes and matrix composition (Kenward et al., 2021). This led to more predictable and reproducible outcomes in therapeutic applications. Moreover, synthetic polymers could be more easily functionalized to improve stability, targeting efficiency, and bioavailability.

PEG is a synthetic, highly water-soluble inert polymer (D’souza and Shegokar, 2016). Its chemical formula is H(OCH_2_CH_2_)n OH, where corresponds to the number of ethylene oxide units (Turecek Peter et al., 2016). Due to their non-toxicity, low immunogenicity, high safety, diverse physicochemical properties, biocompatibility and hydrophilicity, it was commonly used in pharmaceuticals, non-pharmaceutical products, and cosmetics (Yanjie et al., 2024). In 1990, the United States food and drug administration (FDA) approved the first PEGylated protein product for the treatment of severe combined immunodeficiency disease (Jevsevar et al., 2010; Haesun et al., 2022). PEGylation is generally defined as the covalent attachment of activated PEG to therapeutic agents. Since the first PEG modification of bovine serum albumin by Davis in 1977, PEGylation had been widely used in the research and applications to improve the physicochemical properties and biological activities of proteins (peptides), enzymes, antibodies and small molecule drugs. The solubility and flowability of various drugs could be significantly improved by chemical modification with PEG (PEGylation). For example, certain drugs had a short duration of action *in vivo* and were insoluble in water. PEG was utilized in the treatment of drugs to shield them from degradation by the human immune system and extend the drug’s pharmacokinetic profile in the body. Once the drug was treated with PEG, it acted like a layer of “camouflage” to avoid being eliminated by the human immune system as a foreign substance (Veronese and Pasut, 2005). At the same time, PEGylation of drugs reduced the dosage, improved the performance of existing drugs, prolonged the efficacy and reduced the cost of drugs. Therefore, PEG has been widely welcomed in the development of novel nanoparticles for the treatment of hepatic diseases.

PLGA is a biodegradable polymeric nanoparticles. It was approved by the United States. FDA and the European Medicines Agency (EMA) for medical applications, with several formulations such as Decapeptyl, Suprecur MP, and Lupron Depot receiving clinical approval (Makadia and Siegel, 2011). The excellent biocompatibility, biodegradability, and unique physicochemical properties of PLGA made it the most popular and effective drug delivery polymer (Mazen and José, 2022). PLGA was extensively studied for developing delivery vehicles for controlled release of small molecules, proteins, and other macromolecules (e.g., DNA, RNA, and peptides) for commercial (Bouissou et al., 2006; Essa et al., 2020). Since PLGA was biologically hydrolyzed into metabolite monomers (lactic acid and glycolic acid), which were then endogenously metabolized by the body via the citric acid cycle. Therefore, no systemic toxicity was associated with the utilization of PLGA for drug delivery. This positioned it as one of the most attractive polymer candidates in the field of drug delivery systems (Rosli et al., 2021).

PCL is a biodegradable polymer that has been proved to be particularly useful for the controlled and sustained release of drugs due to its slow degradation rate. It might be utilised as a method of encapsulating chemotherapeutic agents for the treatment of liver cancer (Moreira et al., 2022; Zhou et al., 2024). By functionalizing PCL nanoparticles with liver-specific ligands, selective delivery to liver tumors was achieved, enhancing the efficacy of the drugs while minimizing toxicity to healthy tissues (Bate et al., 2022). Additionally, it was metabolized into non-toxic degradation products, ensuring the safety of the microparticles for medical applications.

PMMA, a biocompatible and inertpolymer with high spinnability, is also a hydrophobic polymer (Srikar et al., 2007). It is widely used for the construction of medical devices such as bone cemen, drug delivery applications and microsensorst. It was commonly used for the long-term release of drugs, especially in sustained or extended-release applications. It was employed to encapsulate imaging agents, such as magnetic nano/microparticles or fluorescent dyes, for non-invasive liver cancer detection using MRI, PET, or fluorescence imaging techniques (Montri et al., 2013; Naoki et al., 2019).

Even though various natural and synthetic polymers were developed and tested in recent decades, indicators such as bioavailability, biocompatibility, biodegradability, toxicity, efficiency, and selectivity differed significantly. This was primarily due to the nature of microparticles and their physicochemical properties. The clinical development of the rest of nanoparticles was relatively limited compared to CS, HA, PEG, and PLGA. Currently, there is limited literature available regarding its application as a drug delivery system for liver diseases. Although these nanoparticles had shown promising results in preclinical studies, their clinical translation was slow, often due to challenges such as toxicity, biocompatibility, or difficulties in targeted delivery. In summary, in the following section, the effects of nanoparticles, using CS, HA, PEG, and PLGA as examples, on hepatitis, liver fibrosis, and liver cancer will be reviewed in detail. Hopefully, this review will stimulate further research into liver disease therapies.

## 3 The application of CS in treating hepatic diseases

### 3.1 Delivery of pure bioactive compounds of natural origin and chemical drugs using CS

The study showed that the combination of CS with pure bioactive compounds of natural origin and chemical drugs significantly improved the stability and bioavailability, offering potential benefits for hepatitis treatment while alleviating oxidative stress and inflammatory responses (Metkar et al., 2023). Specifically, it effectively ameliorated ibuprofen-induced elevations in serum aminotransferases, alkaline phosphatase, albumin, and total bilirubin, as well as elevated lipid peroxidation (MDA) and nitric oxide. It also significantly elevated glutathione, glutathione S-transferase, superoxide dismutase, interleukin-6 and B-cell lymphoma-2 (Bcl-2) levels, decreased interleukin-1β and nuclear factor κ-B (NF-κB) levels, enhanced the degradation of toxic effects during ibuprofen treatment, improved the antioxidant system and anti-inflammatory status, as well as increased serum mineral levels (AlKandari et al., 2024).

Compared to the use of drugs alone, combining CS with pure bioactive compounds of natural origin and chemical drugs significantly reduced liver fibrosis. For instance, *Streptomyces malachiticus* coated with curcumin-CS mixture (Elzoheiry et al., 2022), and CS loaded with Itraconazole (ITRCZ) (Elmorsy et al., 2024) or salvinorin B (Sal B) (Yao et al., 2024), could overcome issues of drug insolubility and permeability encountered with single-drug administration. They displayed potent antifibrotic effects, as indicated by the downregulation of transforming growth factor (TGF-β) and tissue inhibitor of metalloproteinases-1 (TIMP-1), as well as decreased hydroxyproline content and α-smooth muscle actin (α-SMA) immunoexpression. They also significantly improved oral bioavailability, while enhancing antioxidant and anti-inflammatory activities.

The pure bioactive compounds of natural origin and chemical drugs using CS for the treatment of liver cancer markedly enhanced the bioavailability and targeting ability of the drugs (Nimeet et al., 2023). Certain factors, such as cell apoptosis and autophagy activation, contributed to liver cancer treatment by modulating cellular processes involved in tumor progression. For instance, CS-coated ivosidenib- PLGA-nanoparticles exhibited higher stability in liver cancer treatment and significantly increased the expression of caspase-3, caspase-9, and p53 (Alsulays et al., 2024). These nanoparticles also inhibited liver cancer cell proliferation, and exhibited anti-migration and anti-angiogenesis properties, while down-regulating Bcl-2 and inducing apoptosis (Harakeh et al., 2023). Curcumin piggybacked niacin-CS could induce autophagy by activating the G-protein coupled receptor 109A (GPR109A)/amp-activated protein kinase (AMPK)/nuclear factor (erythroid-derived 2)-like 2 (Nrf2) signaling pathway, thereby enhancing the cellular degradation (Hong et al., 2017; Hanafy et al., 2023). These nanoparticles effectively targeted subcellular organelles by escaping endosomal and lysosomal cleavage, producing a proton sponge effect. After escaping from the endolysosomal pH, they effectively released rutin into the nucleus, thereby enhancing its cellular bioavailability (Wu et al., 2024). This targeted drug delivery system demonstrated strong performance. Additionally, CS could change the morphology of nanoparticles and improve the stability of nanosystems. The micro/nanoparticles with diameters ranging from 5 to 200 nm contribute to prolonging their circulation time in the bloodstream (Sun et al., 2017; Mondal et al., 2022).

### 3.2 Delivery of vaccine using CS

As an immunostimulant, CS can be used as vaccine adjuvants, improving vaccine efficacy by stimulating specific immunity. CS can induce sustained humoral and cellular immunity against infectious agents or non-infectious diseases. For example, CS has been demonstrated to serve as an effective carrier adjuvant system for interleukin-12 (IL-12) combined with hepatitis B surface antigen (HBsAg), which significantly enhanced HBV-specific CD^8+^ T and CD^4+^ T cell responses, achieving long-term memory against HBV (Zhao et al., 2021). Furthermore, the simultaneous administration of type I IFNs and CS resulted in enhanced serum immunoglobulin G (IgG) and immunoglobulin A (IgA) antibody responses, as well as increased the mucosal IgA antibody response and antitoxin titers (Bento et al., 2019; Maeyama et al., 2020). Meanwhile, nanovaccines were synthesized with precise size distribution and efficient encapsulation through utilizing charge complexation between CS and heparin to encapsulate recombinant hepatitis B virus surface antigen (rHBsAg) or core antigen (rHBcAg) (Qiao et al., 2021).

### 3.3 Delivery of nucleic acids and peptides using CS

CS has gained attention as a non-viral gene delivery system. It could be used as a DNA carrier for gene delivery, as it can condense nucleic acids into stable complexes ranging from 100 to 250 nm in size (Bolhassani et al., 2014). CS-based nanocarriers have been developed for the intracellular delivery of 10–23 DNAzyme, which is assembled into micelles in aqueous solution (Hong et al., 2019). They significantly improved delivery efficiency to hepatocytes and prolonged retention time in the liver (Fatouh et al., 2021). CS nanoparticles demonstrated lower cytotoxicity and high cellular uptake efficiency (Nimesh et al., 2010). Furthermore, CS is often selected as anti-liver fibrosis nucleic acid drug delivery carriers due to its affinity for collagen. CS significantly inhibited collagen over deposition in HSCs and promoted the delivery of anti-TGF-β siRNA through platelet-derived growth factor receptor-β (PDGFR-β) binding peptide modification. *In vivo*, CS successfully accumulated in fibrotic livers via collagen binding peptides (CBPs) modification and inhibited TGF-β1 expression, thus exerting antifibrotic effect (Azzam et al., 2020; El-Safy et al., 2020).

### 3.4 Others

In addition to the three delivery systems mentioned above, CS also demonstrated significant tumor-suppressive effects through the activation of the mitochondrial pathway and endoplasmic reticulum stress, leading to excessive ROS production and subsequent apoptosis in hepatocellular carcinoma cells (Kadry et al., 2018; Jiang et al., 2019). Concurrently, it could be engineered as hydrophilic nanoparticles with a neutral charge to minimize their uptake by phagocytic cells, such as macrophages, within the immune system (Jiang et al., 2022).

## 4 The application of HA in treating hepatic diseases

### 4.1 Delivery of pure bioactive compounds of natural origin and chemical drugs using HA

HA targeted cancer cells by binding to the CD44 receptor, improving drugs delivery efficiency and reducing side effects on normal tissues, thereby enhancing the therapeutic effect (Shao et al., 2024). Studies have shown that self-assembly of methoxy polyethylene glycol-poly lactic acid block copolymer (mPEGPLA) and hyaluronic acid-paclitaxel conjugate (HA-PTX) produced composite nanoparticles (mPPHP NPs) for effective cancer therapy. Paclitaxel (PTX) was rapidly released under the action of HA and esterase enzymes. mPPHP NPs exhibited selective cytotoxicity against A549 cells *in vitro* via CD44 receptor-mediated cellular uptake (Luo et al., 2020).

HA exhibits metabolic targeting. Drug-loaded HA reduced the supply of essential nutrients, such as glutamine and glucose, to cancer cells, thereby enhancing intracellular ROS production. Elevated levels of intracellular ROS trigger the activation of AMPK, resulting in the inhibition of protein kinase B (AKT) phosphorylation and ultimately inducing apoptosis in cancer cells (Ghosh et al., 2023). Varying molecular weights of HA can target solid tumor-associated macrophages, allowing for easy combinations of different components and ensuring that the molecular weight of the polymer or the preparation technique does not adversely affect the targeting ability (Fernandez-Marino et al., 2023).

### 4.2 Delivery of nucleic acids and peptides using HA

RNA interference (RNAi)-mediated gene silencing is frequently employed as an alternative therapeutic strategy for infectious diseases, including refractory hepatitis C virus (HCV) infection. A non-viral vector composed of HA, protamine, and a short hairpin RNA (shRNA74) was designed to target the internal ribosome entry site (IRES) of HCV. This vector demonstrated the capacity to inhibit the expression of the HCV IRES in Huh-7 cells, and it was subsequently rapidly and effectively internalized by the cells (Torrecilla et al., 2016). HA is biocompatible, biodegradable, and has the potential to target tissues and provide receptor-mediated uptake by specific cells, making it a valuable delivery agent for oligonucleotide-based therapies (Rao et al., 2016; Ossipov, 2019).

### 4.3 Others

HA plays a crucial role in the detection and treatment of viral hepatitis. HA-carbon nanotubes (CNTs) composites have been investigated as biofibers or electrode materials for bioelectrochemical applications. Cabral et al. developed a method for the detection of antibodies to hepatitis B core protein (anti-HBc). A nanohybrid surface assembled onto a glassy carbon electrode, consisting of amino-functionalized carbon nanotubes modified with hyaluronic acid, served as a sensing platform for anti-HBc detection. A large number of biomolecules were immobilized on the electrode surface with CNTs membranes, leading to high electron transfer and enabling label-free detection using redox probes (Cabral et al., 2016).

HA has a specific binding affinity for the CD44 receptor, which is highly expressed on the cell surface. During the development of liver fibrosis, activated HSCs (aHSCs) undergo proliferation, leading to a significant increase in CD44 expression on their surface (Kim and Seki, 2023). HA might not only improve drug efficiency but also specifically target aHSCs (Chen et al., 2016; Yu et al., 2024). This targeted approach resulted in the selective induction of apoptosis in aHSCs while sparing quiescent HSCs and hepatocytes (Gong et al., 2023).

The use of HA-coated micelles for drug delivery represents a promising approach for the clinical treatment of liver fibrosis (Li et al., 2020). HA-coated micelles exhibited a core-shell structure comprising a self-assembled biodegradable poly (L-lysine)-b-poly (lactic acid) AB-diblock copolymer (PLys^+^-b-PLLA) core and an exterior coating of HA. The coating was formed through polyion complexation via electrostatic interaction between the anionic HAs and cationic PLys segments. HA-coated micelles exhibited specific cellular uptake of LX-2 cells *in vitro* (Yoshizaki et al., 2023). Furthermore, HA micelles facilitate the increased delivery of chlorosartan, being specifically taken up by HSCs (Thomas et al., 2015).

## 5 The application of PEG in treating hepatic diseases

### 5.1 Delivery of pure bioactive compounds of natural origin and chemical drugs using PEG

PEGylation improved the pharmacological properties of small molecule therapeutics (Hooda et al., 2023). PEGylated interferon (IFN) or nucleoside analogs (lamivudine, adefovir, ribavirin, etc.) were currently important drugs for the treatment of chronic viral hepatitis. Among them, PEGylated IFN-α included PEGylated IFN α-2b (12 kD) and PEGylated IFN α-2a (40 kD), which reduced the clearance of this protein and effectively prolonged the T_1/2_. However, the latter is more stable in blood concentration and had a longer half-life (T_1/2_) due to the greater molecular weight of PEG and better protection against interferon, and its drug distribution was more concentrated (mainly in the blood and liver). PEGylated IFN exhibited a toxicity profile comparable to that of regular α-IFN, while offering a more advantageous pharmacokinetic profile and requiring less frequent administration. Additionally, it enhanced sustained efficacy by approximately 10% relative to regular α-IFN (Tang et al., 2018; Koh et al., 2019; Huang et al., 2022; Etzion et al., 2023; Huang et al., 2023; Wen et al., 2023; Jiang et al., 2024). Meanwhile, PEGylated ribavirin treated patients with chronic HCV infection with an SVR of up to 56% (Abd Ellah Noura et al., 2018; Pawlowski et al., 2024).

Besides, PEGylated drugs could enhance solubility, stability, and pharmacokinetic properties, extending T_1/2_ and reducing side effects (Li et al., 2020). PEGylated IFN α-2a (PEG-IFN-α-2a) (Chen et al., 2021), Pegbelfermin (PGBF), and short-chain PEG-modified curcumin derivative ameliorated the progression of liver fibrosis. These compounds not only increased drug concentrations in the blood but also maintain their anti-inflammatory activity. Meanwhile, they could effectively inhibit the expansion of scar-associated macrophage subpopulations oxidative stress (ROS) and scar-producing myofibroblasts in the damaged liver and remodel the fibrotic ecological niche by modulating ligand-receptor interactions, including platelet-derived growth factor-β (PDGF-β)/platelet-derived growth factor receptor-alpha (PDGFR-α) (Cheng et al., 2018; Xiao et al., 2021). Li F. et al. developed the β-D-Galactose-PEGylated bilirubin nanomedicine (GBRNP) for the treatment of liver fibrosis. It was introduced to target hepatocytes, scavenge ROS. The GBRNP micelles encapsulating selonsertib (Sel@GBRNPs) improved the solubility of the drug and afforded its targeted delivery to hepatocytes.Sel@GBRNPs could synergically protect hepatocytes from H_2_O_2_-induced damage, by decreasing signs of apoptosis and ROS levels. Meanwhile, riociguat combined with Sel@GBRNPs robustly attenuated hepatocyte apoptosis and progression of liver fibrosis in the CCl_4_-induced fibrosis model compared to riociguat + Sel treatment (Li F. et al., 2023).

Pure bioactive compounds of natural origin and chemical drugs loaded in PEG microparticles provided excellent targeted delivery and anti-cancer efficacy for liver cancer (Li et al., 2021). Paclitaxel (PTX) was attached to PEG to form nanodrugs. Due to the oxidation of the core cross-linking structure via sulfhydryl groups, the nanoparticles could be selectively uncoupled by disulfide bond reduction in the reducing microenvironment inside the tumor. This process released the loaded PTX to kill cancer cells while maintaining a high level of stability under physiological conditions outside the tumor (Cai et al., 2021). These nanoparticles were relatively uniform in size, approximately 200 nm, and exhibit a spherical shape. They demonstrated slow-release properties *in vitro*, with a high T_1/2_ and area under the curve (AUC) values, as well as a prolonged circulation time *in vivo*, thus increasing bioavailability of PTX (Qin et al., 2023). The introduction of hydrophilic PEG chain segments into hydrophobic PLGA molecules enhanced their hydrophilic properties, improved stability and anti-enzymatic activities, and enhanced interaction with cellular components. This modification also prolonged circulation time and reduced systemic clearance. The galactosylated chitosan (GC)-coated PEG-PLGA microparticles (GC@NPs) were developed for curcumin (CUR) delivery, which specifically bound to cancer cells that highly expressed the asialoglycoprotein receptor and precisely released CUR. The designed and synthesized microparticles significantly enhanced the drug retention time *in vivo* (Wang et al., 2020; Huang et al., 2023; Huang et al., 2024). Artesunate (ART)-loaded and glycyrrhizin (GA)-modified PEG-PLGA promoted the hepatic targeting distribution of ART, increased retention time, and promoted anti-tumor effects (Pan et al., 2020; Pan et al., 2023). Meanwhile, PEG could promote the anti-tumor effects of natural phenolic antioxidants (Sasaki et al., 2023; Darguzyte et al., 2024). Furthermore, hypoxic tumor cells acted as a key target for treating tumor. For example, PEG with folic acid (FA) and 2-nitroimidazole self-assembled into micelles by chemical coupling, which enabled effective and prolonged drug release in hypoxia-sensitive areas and specifically killed hypoxic tumor cells (Li et al., 2021; Meng et al., 2022). Overall, PEG nanoparticles had been a promising nanocarrier with good biosafety, serving as carriers for the delivery of pure bioactive compounds of natural origin and chemical drugs.

### 5.2 Delivery of nucleic acids and peptides using PEG

As research on PEGylation progresses, its applications are expanding, including as delivery systems for siRNA, mRNA, and pDNA (Wu et al., 2019). No toxicity was detected in an *in vivo* model system using a variety of hepatic cell types and mice with reversed fibrosis. PEG loaded by siRNA was targeted almost exclusively to the liver by intravenous injection and were taken up by nearly 50% of activated HSCs and activated human myofibroblast HSCs (Kaps et al., 2015). PEGylation helps prevent particle aggregation and improves storage stability.

The miRNA nanotherapeutics, activated by a stepwise stimulation of acidity and reduction to mimic the tumor microenvironment, effectively enhanced liver-specific miR-122 expression, thereby increasing the feasibility of translating miR-122 therapy for use against liver cancer (Wu et al., 2019). The PEG has three important novelties: its broad applicability for oligonucleotide cargo delivery, demand-driven pharmacodynamic personalization, and distinguished biodegradability, providing additional possibilities for PEG in the treatment of liver cancer.

### 5.3 Others

Fibrosis hinders polymeric nanoparticles delivery to liver cells, but cationic nanoparticles have been demonstrated to enhance hepatocyte delivery by disrupting endosomal systems and increasing cellular interactions through their pH buffering capacity (Ezhilarasan, 2021). For example, PEG coating enabled nanoscale graphene oxide (NGO) to be predominantly retained in the liver, lungs, and spleen, with particle sizes ranging from 10 to 800 nm, which facilitated the clearance of NGO from these organs, thereby alleviating liver fibrosis (Li et al., 2014). PEG attenuates oxidative stress, the inflammatory response and cellular damage in the liver (Wang et al., 2020; Acar et al., 2022; Bertolini et al., 2024). PEG had good hematological and cytocompatibility, as well as pH-sensitive stability (Xiang et al., 2023). PEG nanoparticles were mixed with extracellular vesicles derived from mesenchymal stem cells (EVs) to form EV-encapsulated PEG hydrogels via a fast, biocompatible click reaction. It could extended the time of EVs targeting in liver fibrosis (Mardpour et al., 2019).

For liver cancer, PEG nanoparticles could utilize redox-responsive components to target cancer cells, and significantly reduce the inflammatory response (Arib et al., 2021; Nisha et al., 2021). More specifically, antioxidants were elevated in hepatocellular carcinoma cells. Therefore, the use of polymers with the anti-oxidant response element allowed them to actively target liver cancer cells and trigger the release of drugs (Powell et al., 2022). Typically, the reduction in blood component interactions induced activation of the complement system, leading to reduced blood clearance of the drug carrier. PEG was a non-ionic hydrophilic polymer that provided a so-called “stealth” coating, which, through spatial stabilization, reduces the tendency of nanoparticles to aggregate and affects the pharmacokinetic properties of the drug or carrier (Cui et al., 2019). PEG shielding or administration extended circulation time and increased the likelihood of the drug reaching the site of action. (Liu et al., 2021; Xiao et al., 2023). The passive targeting of large nanoparticles by the EPR effect represented a pivotal concept in the context of solid tumor targeting in cancer nanomedicine. It was noteworthy that smaller-sized PEGs (≤20 kD, 12 nm) demonstrated substantial tumor targeting with minimal to no nonspecific uptake, whereas larger-sized PEGs (>20 kD, 13 nm) exhibited a marked accumulation in major organs, including the lungs, liver, and pancreas (Kang et al., 2020). PEG microspheres were polymeric particles capable of absorbing organic compounds. They had good compressibility and elasticity and could be suspended for extended periods (Giammaria et al., 2019; Lucatelli et al., 2019). Therefore, it could be attributed to the fact that PEG can persist in liver disease by modulating oxidative stress and the inflammatory response, and control drug release in the form of PEG microspheres.

## 6 The application of PLGA in treating hepatic diseases

### 6.1 Delivery of pure bioactive compounds of natural origin and chemical drugs using PLGA

Herbal extracts from natural medicines have the advantage of low toxicity and fewer side effects. However, the bioavailability and bioactivity of these extracts are limited by poor water solubility and rapid metabolism. PLGA improved the solubility and bioavailability of melatonin, berberine (BBR), and natural metabolites of retinoids (e.g., *all-trans* retinoic acid), and also reduced potential side effects during systemic administration, with good targeting and biosafety. Furthermore, PLGA could be modified to change the structure, packing shape and drug release kinetics of the prepared nanoparticles to meet the needs of different nanomaterial applications (Qiao et al., 2018; Fan et al., 2020; Ji et al., 2020). This approach presents a promising strategy for alleviating liver injury.

Similarly, PLGA could also improve the disadvantages of narrow therapeutic window, severe toxicity, and poor water solubility of antitumor drugs (Li et al., 2024). It was loaded with plumbagin and dihydrotanshinone I (a phenanthraquinone compound of *Salvia miltiorrhiza*) significantly improving the T_1/2_ and tumor targeting of these two drugs in mice with in liver cancer, which yielded the therapeutic effect of reversing the immunosuppressive tumor microenvironment (Han et al., 2022). PLGA loaded with betulinic acid (B) or doxorubicin (DOX) exhibited an initial burst and sustained release (Gao et al., 2019; Kumar et al., 2022). These nanoparticles exhibited stronger cytotoxicity at lower concentrations compared to free drug and were well internalized by HepG2 cells (Upadhyay et al., 2020). Additionally, they were effective in prolonging the T_1/2_ of the drugs, with higher drug concentrations in the plasma and liver. PLGA utilizes EPR effects to passively target tumor, prolong circulation time *in vivo* and improve drug accumulation in the tumor (Wu et al., 2024).

### 6.2 Delivery of vaccine using PLGA

**FIGURE 3:**
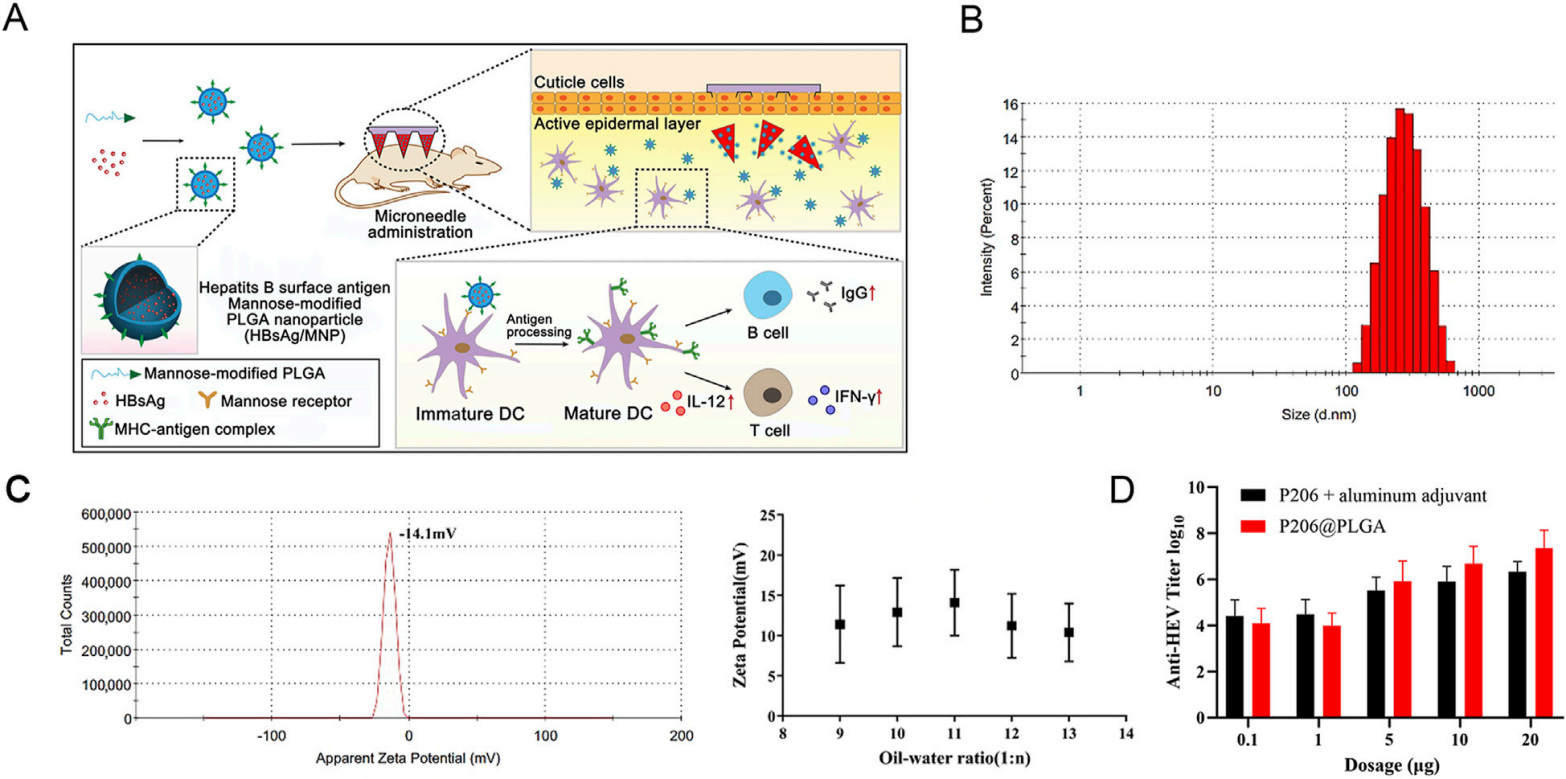
**(A)** The structure of PLGA nanoparticles and the immune response for nanoparticles *in vivo*. Reprinted with permission from (Zheng et al., 2023). Copyright 2024, Springer Nature. **(B)** P206@PLGA nanoparticles size and **(C)** zeta potential. **(D)** Determination of anti-HEV antibody titer in the serum after immunization with P206 plus aluminum adjuvant and P206@PLGA. The antibody titer induced by P206@PLGA in the 20 µg group was significantly higher than that in the P206 plus aluminum adjuvant group (p < 0.05). Reprinted with permission from (Yang et al., 2022). Copyright 2022, MDPI.

PLGA has the capability to encapsulate various antigens and deliver them to antigen-presenting cells (APCs) (Figure 3A) (Zheng et al., 2023). PLGA-based vaccines have been demonstrated to effectively target APCs, thereby inducing robust cellular and humoral immune responses (Zhang et al., 2019). As an adjuvant for a therapeutic vaccine against viral hepatitis, PLGA significantly induced secretion levels of IL-2 and IFN-γ, and increased the absolute number of CD^4+^ and CD^8+^ T cells, triggering a cell-mediated immune response in mice (Roopngam et al., 2016; Mohammed and Naif, 2022). Additionally, it can effectively reduce adverse reactions associated with vaccinations and lower the required dosage in immunized animals, thereby improving the overall immune effects (Allahyari and Mohit, 2016).

Moreover, PLGA was applied as a nanocarrier to encapsulate P206 (the immunogenic ecombinant protein), resulting in the preparation of P206@PLGA microparticles with a particle size of approximately 354 nm and a negatively charged surface. It was uniform in size, stable in quality, and could improve the bioavailability and prolong the duration of drug action. P206@PLGA was found to be more stable and induced higher antibody titers compared to traditional vaccines containing P206 and aluminum adjuvant (Figures 3B–D) (Yang et al., 2022). Once microparticles entered the body, they were rapidly engulfed by macrophages of the reticuloendothelial system, resulting in expedited removal from the circulation. This was observed within 30 min following intravenous administration. A substantial quantity (60%–90%) of antigen-loaded particles were subsequently distributed to the liver (Dai et al., 2017; Dewangan et al., 2018).

Intravenous administration of PLGA nanovaccines [PLGA loaded with ovalbumin and invariant natural killer T (iNKT1) cell agonist] mainly targets the liver and spleen where iNKT1 cells are abundant and result in the highest serum IFN-γ levels, T cell cytotoxicity, and Th-1 type antibody responses (Dolen et al., 2020). This offers insights into the development of vaccines for anti-tumor applications.

### 6.3 Delivery of nucleic acids and peptides using PLGA

PLGA has also received widespread attention for delivering nucleic acids and proteins in the treatment of liver cancer. The small interfering RNA (siRNA) targeting alpha-fetoprotein (AFP) mRNA was found to induce apoptosis and cell death in HepG2 cells, highlighting its potential as a therapeutic agent. To deliver the AFP siRNAs into liver cancer cells, the siRNAs were loaded into the nanoparticles based on PLGA. It provided a sustained release of the drug, thereby maintaining high drug concentration at the target site while reducing systemic toxicity (Pho-Iam et al., 2021).

### 6.4 Others

The profibrogenic liver environment, characterized by fibrogenesis and chronic stimulation of HSCs, is formed by hypoxia and hydrogen peroxide (H_2_O_2_) accumulation. PLGA exhibits enhanced antifibrotic efficacy by eliminating excess H_2_O_2_ and alleviating hypoxic stress (Dong et al., 2023). This makes PLGA particularly relevant in the context of liver disease treatment. Choi et al. designed CO_2_ gas generating PLGA microsphere (MS) for the rapid release of tunicamycin in TACE treatment for liver cancer (Choi et al., 2021). Drugs were dispersed or dissolved in MSs and released slowly through various mechanisms such as self-diffusion, biodegradation, dissolution and osmotic pressure, which improved the efficacy and reduced side effects. Commonly used carrier materials include poly (lactic acid), poly (glycolic acid), and PLGA. PLGA is a co-polymer constituted by lactic acid and glycolic acid at a certain proportion. The hydrophilicity and degradation cycle of PLGA could be controlled by adjusting the molecular weight and the lactic-to-glycolic acid ratio, thereby optimizing their performance in drug delivery applications (Tian et al., 2018). In recent years, PLGA MSs have received widespread attention due to their excellent biocompatibility and biodegradability.

Biodegradable block copolymers play a crucial role in the field of nanoparticles, particularly for drug delivery applications. This is due to their efficient drug loading rate, good biocompatibility, and high bioavailability. Among them, PLGA-PEG is currently widely used (Mo et al., 2022; Lin et al., 2024). PLGA-PEG demonstrated good loading efficiency for BEZ235 (a PI3K/mTOR inhibitor) and high selectivity for Glypican-3-positive HepG2 cells (Tang et al., 2020). At the nominal concentration, they synergistically killed liver cancer cells with significantly higher efficiency than the free drugs. These characteristics make PLGA-PEG a promising candidate for improving the therapeutic efficacy of anticancer treatments.

## 7 Discussion

Hepatic diseases pose a serious threat to human health. The progression from hepatitis to liver fibrosis to liver cancer is also known as the “liver cancer trilogy”. Currently, drugs such as interferon, certain pure bioactive compounds of natural origin, sorafenib, and lenvatinib are primary treatment strategies for hepatic diseases in clinical practice. However, their use is limited due to low bioavailability, poor solubility, and the potential for drug resistance, and various side effects associated with long-term use. Liver transplantation is a successful treatment method for advanced liver cancer patients, yet it faces challenges such as organ shortages and high medical costs. In recent years, new drugs for liver disease have been developed continuously to improve the bioavailability and efficacy of delivered drugs. Polymeric nanoparticles offer many advantages as drug delivery systems, including regulated size and shape, highly specific targeting, stimuli responsiveness and high loading capacity (Patra et al., 2018). As an interdisciplinary field, nanotechnology has been widely used in researching liver disease treatment and has demonstrated promising therapeutic effects. Although well-appreciated results have been achieved, there are still many issues to be resolved.

First, Nanoparticles are more susceptible to aggregation, hygroscopic properties, contamination and degradation because of their high surface area and large nanoscale dimensions. Inappropriate storage conditions may lead to aggregation or degradation, necessitating rigorous standards for storage conditions and durations of nanomedicines (Muthu and Feng, 2009). Additionally, the preparation process for some polymeric nanoparticles is complex and has poor repeatability, making some nanoparticles difficult for large-scale production for clinical applications (Shepherd et al., 2021). Furthermore, the targeting and therapeutic efficacy of polymers is significantly constrained by the high complexity of both the liver and the cancer microenvironment. Since endogenous signals inside and outside cancer cells are difficult to control and vary between individuals, this variability can lead to erratic therapeutic effects. Several solutions address these issues. First, introducing microfluidic devices is one approach to solving the problem. It improves the scalability of polymeric nanoparticles and reduces production time and preparation steps. Additionally, it enables uniform control over the properties of drugs on a larger scale, which reduces the risk of aggregation or degradation (Valencia et al., 2020). Secondly, simple, rapid and affordable high-throughput screening methods can be employed to optimize various formulation parameters of nanoparticles. The design of experiments (DOE) is one of the most promising strategies (Jones et al., 2016; Ho et al., 2019). Furthermore, new manufacturing techniques and equipment for nanoparticles are being developed to produce low-toxicity polymeric nanoparticles, while simplified preparation processes facilitate large-scale production. Nanocarrier systems can be designed to contain multiple targeting mechanisms. Unlike single targeting approaches, these nanoparticles can carry two or more “guidance' signals simultaneously, increasing the accuracy and probability of identifying targeted tissue. Finally, it is also important to develop biomimetic *in vitro* test platforms and preclinical models that can effectively evaluate the properties of polymeric nanoparticles. Such assessments can more accurately predict their behavior *in vivo*. In the future, researchers may be able to provide personalized and precise drug delivery for patients by understanding the immunology of liver disease. For example, polymeric nanoparticles can target macrophages in the liver and improve the efficacy of treatments by modulating their immune response (Tacke, 2017). A rigorous biosafety assessment is required for polymeric nanoparticles, evaluating immunogenicity, the safety of degradation products, effects on liver function, and pharmacokinetic effects on the drug delivery system. Although several polymers for hepatic diseases are currently in clinical trials (Table 2), further efforts are needed to develop effective treatments for hepatic diseases. Therefore, it is essential to develop nanoparticles suitable for liver disease treatment and to further study the mechanisms of nanoparticles in hepatic diseases. Natural polymers and synthetic polymers each presented unique advantages and challenges in the treatment of hepatic diseases. Natural polymers excelled in biocompatibility and biodegradability, making them ideal for long-term therapies, while synthetic polymers offered greater control over drug release and targeting, making them suitable for precision medicine applications. The development of hybrid nanoparticle systems that combined the best features of both types of nanoparticles might hold the key to more effective and safer treatments for hepatic diseases in the future.

**TABLE 2:** List of clinical trials of polymers for hepatic diseases (clinicaltrials.gov).

Conditions	Name	Carriers	Functional component	Clinical trialsidentifier	Phase	StartYears	Status	Sponsor
HBV	PEG-IFN α-2b	PEG	IFN α-2b	NCT01928511	Phase 4	2014–01	Completed	Seng Gee Lim
HBV	IFN + resveratrol	PEG	IFN	NCT03546530	-	2016–06	Completed	First Hospital of Jilin University
HBV	REP 2139-Mg	PEG	IFN	NCT02565719	Phase 2	2016–03	Completed	REPLICor Inc.
Chronic Hepatitis, HBV	Peginterferon α-2a	PEG	IFNα-2a	NCT02992704	Phase 2, Phase 3	2016–08	Unknown	Seng Gee Lim
HBV	PEG-IFN α-2b	PEG	IFN α-2b	NCT05451420	Not Applicable	2017–12	Completed	Henan Provincial People’s Hospital
HBV	HBIg	PEG	Hepatitis B immune globulin, IFN	NCT03575208	Phase 2	2019–02	Withdrawn	National Institute of Diabetes and Digestive and Kidney Diseases
HBV	PEG-IFN	PEG	IFN	NCT05357235	-	2022–01	Recruiting	Beijing Ditan Hospital
HBV	PEG-IFNα-2b, Nucleoside Analogs	PEG	α-2b, nucleoside analogs	NCT05182463	Phase 4	2022–01	Recruiting	Third Affiliated Hospital, Sun Yat-Sen University
HDV	Bulevirtide	PEG	IFN α-2a	NCT03852433	Phase 2	2019–05	Completed	Gilead Sciences Inc.; MYR GmbH
Liver fibrosis	Entecavir	PEG	Entecavir, IFN	NCT02849132	Phase 4	2016–01	Active, not recruiting	Beijing Friendship Hospital
Liver cancer	weGePM-HCC	Polymeric micelle	Paclitaxel	NCT03008512	Phase 2	2016–10	Terminated	Gachon University Gil Medical Center
Liver cancer	HEPA-China-002	Polymer microsphere	Doxorubicin	NCT02743065	-	2016–09	Unknown	Fifth Affiliated Hospital, Sun Yat-Sen University

HBV, hepatitis B virus; IFN, interferon; HBIg, hepatitis B immune globulin; HDV, hepatitis D virus.

In conclusion, this review discussed the advantages of natural polymers and synthetic polymers as drug delivery systems. Furthermore, the paper focused specifically on CS, HA, PEG and PLGA in the treatment of hepatitis (particularly viral hepatitis), liver fibrosis, and liver cancer, with the aim of exploring new strategies for the treatment of hepatic diseases. It also discussed various issues and future research directions. Despite the progress made in this field, significant challenges remain. Addressing these issues through targeted research is crucial for developing more cost-effective and practical nanomaterials that can enhance the clinical implementation of treatments for hepatic diseases. By focusing on these advancements, future studies can contribute to improved patient outcomes and more effective therapeutic options.
